# Simultaneous Measurements of Structure and Water Permeability in an Isolated Human Skin Stratum Corneum Sheet

**DOI:** 10.3390/polym11050829

**Published:** 2019-05-08

**Authors:** Hiromitsu Nakazawa, Tomohiro Imai, Mika Suzuki, Natsuki Akakabe, Ichiro Hatta, Satoru Kato

**Affiliations:** 1 School of Science and Technology, Kwansei Gakuin University, Sanda 669-1337, Japan; imai.tomohiro.01@gmail.com (T.I.); miica4-e@yahoo.co.jp (M.S.); ean82173@kwansei.ac.jp (N.A.); sk@kwansei.ac.jp (S.K.); 2 Department of Research, Nagoya Industrial Science Research Institute, Nagoya 460-0008, Japan; hatta@pj9.so-net.ne.jp

**Keywords:** intercellular lipid, stratum corneum, transepidermal water loss, wide-angle X-ray diffraction

## Abstract

Stratum corneum (SC), the outermost layer of human skin, acts as an intelligent physicochemical interface between the inside and the outside of our body. To make clear the relationship between structure and physical barrier properties of SC, we developed a method that enables us to simultaneously acquire X-ray diffraction (XD) patterns and transepidermal water loss (TEWL) values using a spread SC sheet isolated from human skin. The synchrotron X-ray was incident on the SC sheet surface at an angle of 45° to avoid interference between the two kinds of measurements. Detailed comparison between XD and TEWL data suggested that the thermal behavior of water permeability is closely related to the thermal expansion of the lattice spacings of the hexagonal phases above 40 °C and to the existence ratio of the orthorhombic phase below 40 °C. Thus, the new method we developed can give useful information on the mechanism of water permeation in SC without ambiguity caused by separate measurements of structure and water permeability with different samples.

## 1. Introduction

The outermost surface layer of the human skin is the stratum corneum (SC), which serves as a physicochemical barrier between inner body and outer environment. The SC is mainly composed of dead epidermal cells (corneocytes) embedded in an intercellular lipid-rich matrix, providing our body with a highly sophisticated barrier against not only excess water evaporation from inner body but also penetration of foreign substances from outside [1,2,3]. Recent X-ray, neutron, and electron diffraction studies have revealed that intercellular lipids are organized in multi-lamellar structures with repeat distances of about 6 nm and 13 nm, and arranged in orthorhombic (Ort) and hexagonal (Hex) lattices within the lamellae in healthy SC [4,5,6,7,8,9,10]. Moreover, in addition to these two kinds of ordered phases, FTIR studies have indicated that a small amount of disordered liquid (Liq) phase coexists with the Ort and Hex phases [11].

The water content in the whole SC of healthy skin has been estimated to be about 20–30% *w*/*w* (weight of water/weight of SC) [12,13]. Water is considered to be constantly replaced in SC. Namely, the water content of 20–30% *w*/*w* is maintained by balancing the inflow from viable epidermis with evaporation from the skin surface [14]. As the extent of water evaporation from SC is quantified as transepidermal water loss (TEWL), a TEWL sensor is usually used to evaluate the barrier function of skin [15,16,17].

Generally, impairment of the barrier function of SC reduces the SC ability to prevent water from over-evaporating, resulting in an increase in TEWL. Recent studies have revealed that the organization of intercellular lipids plays a crucial role for the skin barrier [18,19]. For example, Pilgram et al. reported that the Hex-to-Ort ratio in SC as well as TEWL is higher in the atopic dermatitis and lamellar ichthyosis skins than in the healthy skin, suggesting that the Ort phase is more water-tight than the Hex phase [20]. However, as well as we know, there is no unequivocal quantitative evidence showing the causality between lipid organization in the intercellular matrix and TEWL. Direct evidence should be required to elucidate the role of intercellular lipid organization for the skin barrier properties.

In this study, we measured temperature dependence of the X-ray diffraction (XD) pattern and TEWL in the isolated human SC to clarify how the lipid organization affects the water permeability. These two kinds of measurements were carried out simultaneously using the same SC sample because the component and structure of SC are slightly different from preparation to preparation and even in the same sample its physicochemical state is affected by various factors such as preparation process, temperature hysteresis, and hydration level [6,21,22]. For this purpose, we newly developed a sample holder for simultaneous measurements of structure and water permeability in an isolated human SC sheet. Detailed comparison of XD and TEWL data made it possible to evaluate the effects of fine structural changes on the water permeation processes.

## 2. Materials and Methods

### 2.1. Preparation of SC Samples

Sheets of human SC were purchased from Biopredic International (Rennes, France). The SC sheet had been isolated from breast skin immediately after cosmetic surgery according to the French law L. 1245 CSP “product and element of human body taken during surgical procedure and used for scientific research”. The procedure of SC isolation from skin has been described elsewhere (Bouwstra et al., 1992) [23]. The SC sample was dried and stored in a vacuum desiccator connected to a rotary pump until use. The SC sample was hydrated to 25% *w*/*w* (weight of water/weight of hydrated SC) at room temperature for two days before the experiment in order to be equilibrated.

### 2.2. Sample Holder for Simultaneous Measurements of SC Structure and Water Permeability

**Figure 1:**
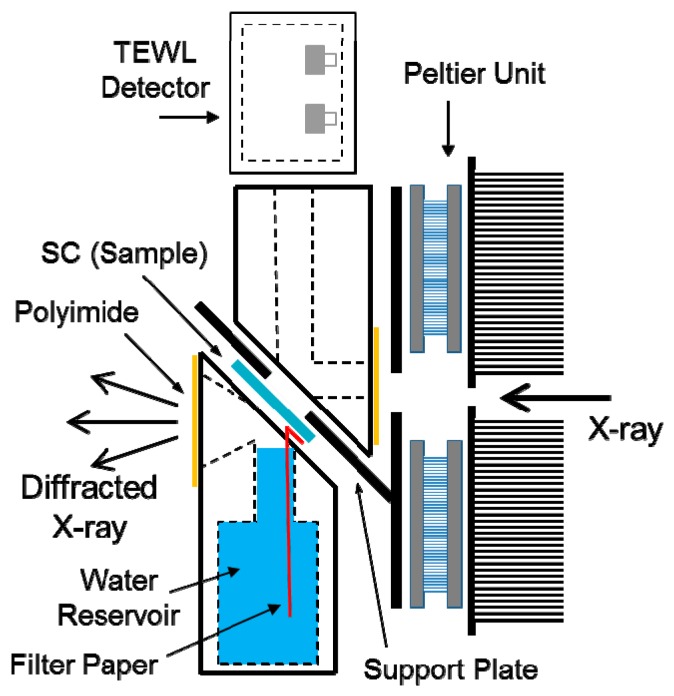
Schematic illustration of the new sample holder designed for simultaneous measurements of the structure and function of a sheet-like sample. The sample is closely attached to the support plate with a small hole at 45° from the direction of the incident X-ray beam and sandwiched between two separable chambers, whose inner environments can be controlled independently. A transepidermal water loss (TEWL) sensor (Tewameter) is attached on the top of the upper chamber to measure water permeability during the X-ray diffraction measurement. Water is filled in the lower chamber and supplied to the underside of the sample through a piece of paper. The position of the upper chamber is slightly shifted in the illustration for the sake of simplicity.

A schematic illustration of the newly developed sample holder is shown in Figure 1. The sample support plate with a hole was tilted at about 45° with respect to the direction of the incident X-ray beam, and its temperature was controlled by a Peltier element with a heat sink directly connected to it. A sheet-like sample could be easily placed on the support plate by removing the upper block from the lower blocks. A vertical path for diffusing water molecules with a diameter of about 4 mm and a horizontal path for the X-ray beam with a diameter of about 2 mm were arranged mutually perpendicular as indicated by dotted lines in Figure 1. To avoid dehydration of the sample, both ends of the horizontal path were sealed with thin polyimide films. A TEWL (transepidermal water loss) meter was set on the top of the upper block.

### 2.3. Synchrotron X-ray Diffraction

Synchrotron X-ray diffraction experiments with the sample holder described above were performed at BL03XU and BL40B2 in SPring-8 (Hyogo, Japan). The details of these beamlines have been described elsewhere [24,25]. The energy of X-ray was 14 keV, which corresponds to a wavelength of 0.0886 nm, and the sample-to-detector distance was about 550 mm. The cross section of the X-ray beam at the sample position was approximately 0.2 × 0.2 mm^2^ and the exposure time was about 30 s. The two-dimensional imaging plate detector R-AXIS VII (RIGAKU, Tokyo, Japan) with a detection area of 300 × 300 mm^2^ and the semiconductor detector Pilatus 1M (Dectris, Baden, Switzerland) with a detection area of 169 × 179 mm^2^ were used for data collection. The reciprocal spacing *s* = (2/*λ*) sin (2*θ*/2), where 2*θ* and *λ* are the scattering angle and the wavelength, respectively, was calibrated using the lamellar spacing (*d* = 3.39 nm) of anhydrous cholesterol at room temperature.

To analyze the thermal behavior of wide-angle X-ray diffraction (WAXD) profiles, the obtained diffraction pattern was circular-averaged using an original program to obtain a radial intensity profile. If necessary, the background was subtracted using an intensity profile of water filled in the sample holder after normalization at an appropriate s value. The deconvolution of the peak was performed by fitting the data to Lorentzian functions using the simplex method. 

### 2.4. Simultaneous Measurements of Structure and Water Permeability in SC

An SC-sheet sample was spread over the sample support plate and fixed at the upper and lower ends of the sample. The water permeability was measured with Tewameter^®^ (Courage+Khazaka, Köln, Germany) with a resolution of 0.5 gh^−1^m^−2^ attached to the upper part (acceptor chamber of diffusant) of the sample holder (see Figure 1). This device is based on the diffusion principle in an open chamber (see [26] for details). For the TEWL measurement, water was placed into the reservoir (donor chamber of diffusant) in the sample holder not so as to disturb the passing of scattered X-ray and supplied to the marginal part of the underside of the SC sheet with a bridging paper. The temperature of the sample was monitored with a K-type thermocouple attached to the sample support plate in the neighborhood of the sample.

Water permeability was measured every 10 s and smoothed by the moving average method. The X-ray diffraction pattern was acquired every 180 s (R-AXIS VII) with an exposure time of about 30 s, or 30 s (Pilatus 1M) with an exposure time of about 15 s. As temperature was scanned at a rate of 0.6 K/min both in heating and cooling, the TEWL value and the X-ray diffraction pattern were obtained every 0.1 °C and every 1.8 °C (R-AXIS VII)/0.3 °C (Pilatus 1M), respectively. The control experiment for TEWL was carried out using a hydrophilic Millipore GS-type membrane filter instead of the SC sample.

## 3. Results

### 3.1. Development of a Method for Simultaneous Analysis of the SC Structure and Function

We tried to develop a new method which allowed the simultaneous measurement of an indicator related to substance permeability during a dynamic X-ray diffraction measurement to obtain clear-cut evidence for correlation between the lipid packing structure and barrier function in the skin stratum corneum (SC). For this purpose, we designed a sample holder whose detailed layout is explained in the Materials and Methods section. The key point of the holder design is how to avoid the interference between the two types of measurements. We solved this problem by setting a spread SC sheet on the sample support plate with a hole tilted at 45° with respect to the incident X-ray beam.

**Figure 2:**
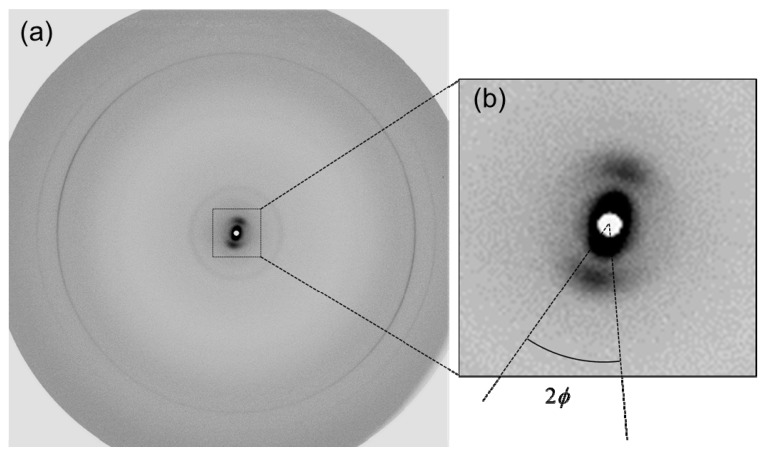
An X-ray diffraction pattern of an isolated human stratum corneum (SC) sheet obtained using the sample holder described in (**a**) and the enlarged image of the small angle region (**b**). Angular spread of the reflection corresponds to the extent of the orientation of the intercellular lipid layers (see text).

The X-ray diffraction pattern we obtained using the newly developed sample holder exhibited arc-like reflections as expected from the structural anisotropy of the SC sheet (Figure 2). Since lipid layers oriented in parallel with the average surface of the SC sample give no reflection of the lamellar structure in the present experimental geometry, they must be undulated as seen in electron micrographs of the SC thin sections stained by ruthenium tetroxide [27,28]. As the half central angle of the clear reflection arc in the small angle region was roughly 20°, for the lipid layers which gave the reflection the range of the tilt angle from the average SC surface was roughly estimated to be 45–48° according to the equation derived from a simple geometrical consideration; cos*α* = cos*θ* cos*ϕ*, where *α*, *θ*, and *ϕ* are the maximum tilt angle of the lipid layer, the tilt angle of the sample support plate (45°) and the half central angle of the arc, respectively. Here, we assumed that the deviation of the symmetry axis in the diffraction pattern from the vertical direction was caused by the bending of the SC sheet in the hole of the sample support plate.

We used a stack of plural SC sheets as a sample to obtain the data in Figure 2 because the reflection from a single SC sheet in the small angle region was too weak to obtain an intensity sufficient for reliable analysis. Hence, we focused only on temperature dependence of the wide-angle region in the experiment below, where a single SC sheet was used as a sample to simultaneously acquire X-ray diffraction patterns and TEWL values.

### 3.2. Temperature Dependence of Water Permeability

Temperature dependence of water permeability of the SC sheet was examined with a TEWL sensor (Tewameter) set on the sample holder (see Figure 1). Here, we call the water permeability detected by the Tewameter “TEWL” (transepidermal water loss) according to the conventional terminology and, for convenience, the Tewameter output value without any correction was plotted as the TEWL value.

**Figure 3:**
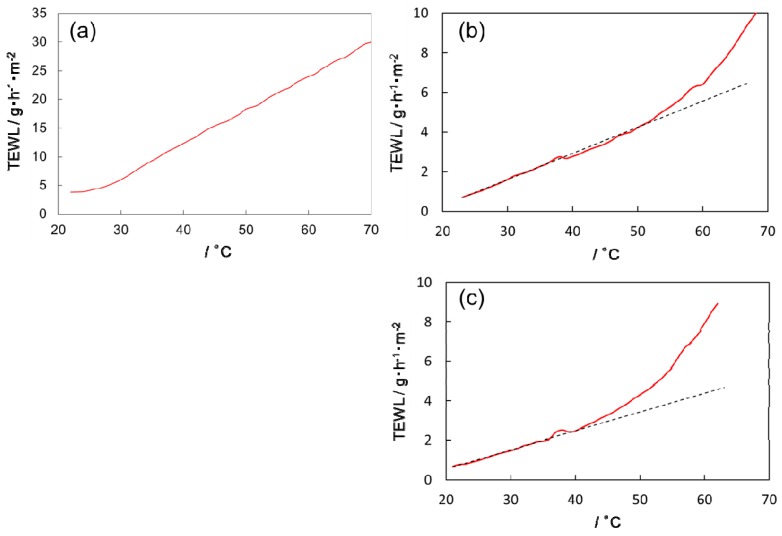
Temperature dependence of TEWL in (**a**) a membrane filter in heating, (**b**) an SC sheet in heating, and (**c**) an SC sheet in cooling. The output value of Tewameter without correction is plotted as the TEWL (transepidermal water loss) value. The scanning rate of temperature was 0.6 K/min. The data was acquired every 10 s and smoothed by the moving average method. The dashed lines in (**b**,**c**) are calculated by linear regression of the data at low temperature region and shown as a guide for eyes.

The TEWL value in the SC sheet was much lower than that in the membrane filter at every temperature measured (Figure 3), indicating that difference in water permeability across thin samples is detectable in the present experimental setting. Furthermore, the manner of temperature dependence of TEWL in the SC sheet was different from that in the membrane filter; the overall dependence seemed non-linear in the former and almost linear in the latter. 

In the case of the SC sample we can identify three temperature regions divided by stepwise changes which often appeared at ~39 °C and ~60 °C in heating, and at ~57 °C and ~39 °C in cooling. The trend of the change in TEWL in the two higher temperature regions seemed to be continuous, showing a non-linear increase with increasing temperature in spite of the stepwise change between these two regions. In contrast, the TEWL value in the lowest temperature region seemed to increase linearly as the overall TEWL value of the membrane filter. These changes in the diffusion mode as well as the temperature hysteresis of TEWL coincided well with the behavior of structural changes observed by simultaneously measured XD (see below). 

### 3.3. Thermal Behavior of the Lipid Organization in SC

**Figure 4:**
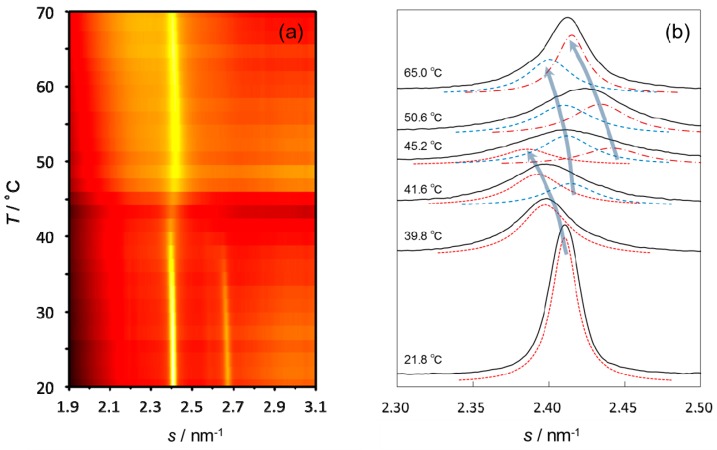
Thermal behavior of the wide-angle X-ray diffraction (WAXD) profiles of human SC in heating from 21.8 °C to 70 °C. (**a**) The temperature dependence of the intensity profile is shown in a colored map (in the order of black < dark red < red < yellow). The scanning rate of temperature was 0.6 K/min, and the data was acquired every 180 s. Two sharp peaks at ~2.4 nm^−1^ (P_2.4_) and ~2.7 nm^−1^ (P_2.7_) are clearly seen in low temperatures. (**b**) Deconvolution of the peak P_2.4_. The intensity profile (solid line) was fitted to Lorentzian function(s) by the simplex method so that every deconvoluted peak (dotted line) shows thermal expansion. Arrows are drawn to guide the eye.

Figure 4a shows temperature dependence of the XD intensity profile of the SC sheet obtained simultaneously with the TEWL measurement in heating from 21.8 °C to 70.0 °C. Overall thermal behavior of the intensity profile was similar to that in the experiment using an SC sample randomly folded and stacked in a sample cell [22,29]: At room temperature the WAXD profile exhibited two sharp peaks at around *s* = 2.4 nm^−1^ and *s* = 2.7 nm^−1^ resulting from coexistence of orthorhombic (Ort) and hexagonal (Hex) phases. Here, for convenience, we designate these two peaks P_2.4_ and P_2.7_, respectively. As temperature increased, these two peaks transformed into a broader peak with the s value slightly higher than that of the initial P_2.4_. However, we noticed that some of the broad peaks appearing after the disappearance of P_2.7_ were evidently asymmetric as shown in Figure 4b. This means that plural hexagonal phases with different lattice constants coexist in higher temperatures. Therefore, we tried to deconvolute the broad peak by fitting the data to two or three Lorentzian functions so that each peak shifted to a smaller angle with increasing temperature (dashed lines in Figure 4b).

**Figure 5:**
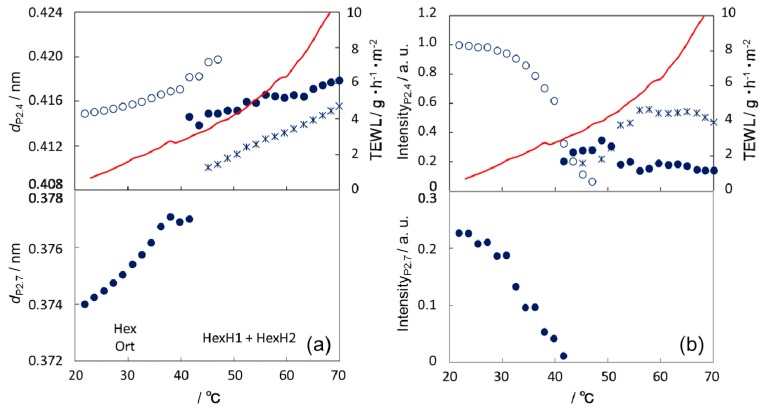
Comparison of TEWL with WAXD of human SC in heating. (**a**) Lattice spacings *d* calculated from the positions of P_2.7_ (●) and deconvoluted peak(s) (◌, ×, and ●) as described in Figure 4b and (**b**) their peak intensities are shown as functions of temperature, together with TEWL data shown in Figure 3b.

Figure 5 shows temperature dependence of the lattice spacings and integrated intensities of P_2.4_ and P_2.7_ in heating, together with that of TEWL measured simultaneously. The stepwise change of TEWL at ~40 °C agrees with disappearance of the Ort phase or appearance of new hexagonal phases (we designate these new phases as HexH1 (●) and HexH2 (×)). The other stepwise change at ~60 °C took place at the lower temperature end of the plateau region of integrated intensities of HexH1 and HexH2. Thus, these stepwise changes of TEWL may be related to the beginning of a new structural state.

Interestingly, the lattice spacings of HexH1 and HexH2 increased almost linearly with increasing temperature, irrespective of the variation of their existence ratio, and this continuity of change in the lattice spacings coincided with the thermal behavior of TEWL in the relevant temperature region.

**Figure 6:**
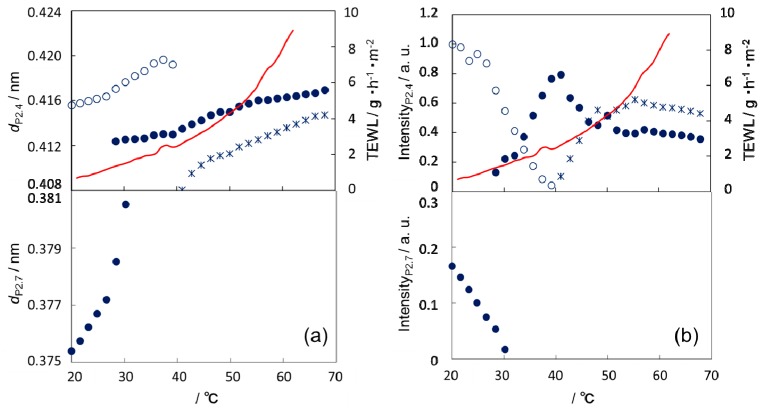
Comparison of TEWL with WAXD of human SC in cooling. (**a**) Lattice spacings calculated from the positions of P_2.7_ (●) and deconvoluted peak(s) (◌, ×, and ●) as described in Figure 4b and (**b**) their peak intensities are shown as functions of temperature, together with TEWL data shown in Figure 3c.

Similar correspondence in the relationship between XD and TEWL data was also observed in cooling (Figure 6) though disappearance of HexH2 and appearance of the Hex phase rather than the Ort phase agreed with the stepwise change of TEWL at ~40 °C. The lipid packing structures at the same temperature in heating and cooling were evidently different, suggesting that the SC structure has a large temperature hysteresis and is in a non-equilibrium state. This means that the structure at a certain temperature depends on the history of the preparation and is one of the reasons why simultaneous measurements of structure and TEWL are favorable. 

## 4. Discussion

We succeeded in acquiring X-ray diffraction (XD) patterns and TEWL values simultaneously in human skin stratum corneum (SC) during a temperature scan and obtained a direct evidence for the relationship between the lipid organization in SC and the skin barrier function. The new method we developed in this study provides the fundamental layout to measure additional physicochemical parameters during X-ray diffraction measurements. We designed a new sample holder which holds a single spread SC sheet between donor and acceptor chambers for the diffusant molecule at an angle of 45° with respect to the incident synchrotron X-ray beam (Figure 1). The slanted SC sheet was able to give Bragg reflections strong enough for quantitative analysis in the wide-angle region. We did not take the undulation state of lipid layers into account in the interpretation of the reflection intensity, assuming that the undulation changed little due to the high bending energy of the ordered phases dominant in the temperature range measured. 

The water permeability of the SC sheet was evaluated with an open-chamber type TEWL sensor (Tewameter) placed on the top of the acceptor chamber (Figure 1). In the Results section, we showed the results in which the TEWL values were significantly lower than those for the membrane filter and exhibited similar thermal behavior in the heating and cooling scans. In some cases, however, the TEWL value increased almost linearly as temperature increased as in the filter membrane probably because the SC sheet adhered poorly to the sample support plate. In addition, we plotted TEWL as relative values because it was hard to obtain reliable absolute values due to indefinite effective area of the sample, inhomogeneous temperature distribution and water condensation in the acceptor chamber. Although further improvement is required in the TEWL measurement to obtain more suitable data for quantitative analysis, the relative TEWL data qualitatively reflect the state of water permeation in the SC.

In this study we showed only the best data to show the correlation between SC structures and water permeability because the reproducibility of TEWL data was low and the scope of this study is limited to establishing the new method for acquiring two kinds of data simultaneously using a spread SC sheet. As it is known that structures and physicochemical properties of SC significantly vary from sample to sample, a subject for future studies will be the detailed analysis of the difference of correlation pattern between samples.

Comparison of TEWL and XD data provides an insight into the mechanism of water diffusion in SC. It suggested that the temperature dependence of TEWL showed two stepwise changes at the beginning of structural transitions. In heating new hexagonal phases, HexH1 and HexH2, started to form at ~40 °C (Figure 5) and the fluid phase might start to form at ~60 °C as described previously [23,29]. We speculate that the stepwise change is related to the structural change at the boundary between structural domains with different orientations, considering that the phase transition may start from the domain boundary, which is a plausible pathway of water diffusion due to lattice defects. The subsequent transition inside the domain may have a much smaller effect on TEWL because it may hardly affect the orientation (direction of the symmetry axis of lipid lateral packing) of the domain. If it is the case, the water diffusion through the defects at the domain boundary may cause only a secondary effect (the extent of the stepwise change in TEWL is relatively small). 

The fact that the nonlinear increase in TEWL above 40 °C except for the stepwise change around 60 °C occurred in parallel with the linear increase (thermal areal expansion) in lattice spacings of HexH1 and HexH2 suggests that the lateral molecular packing density in the intercellular lipid layer may be the key factor for the water diffusion in SC. In the temperature range from 40 °C to 60 °C variation of the ratio between hexagonal phases with different lattice spacings makes the situation more complicated. Further discussion should be postponed until more information on the properties of each hexagonal phase is available. Incidentally, it should be noted that temperature dependence of diffusion constant may not be the dominant factor to determine the thermal behavior of TEWL because the overall temperature dependence of TEWL was not the Arrhenius type with a constant activation energy.

In contrast, the increase in TEWL below 40 °C seemed linear, suggesting that the determinant factor for the thermal behavior of TEWL may be different from that above 40 °C. The structural characteristic below 40 °C is definitely different from that above 40 °C and consists of the coexistence of the Ort phase with high lateral packing density and the Hex phase with low lateral packing density. If the lateral packing density is the key factor for water diffusion in SC as discussed above, the Ort phase must be a much higher barrier against water permeation than the Hex phase. Therefore, we infer that the existence ratio of the Ort phase rather than the thermal areal expansion may determine the thermal behavior of TEWL below 40 °C.

In summary, we speculate that the thermal areal expansion of the hexagonal phase may give nonlinearity to the thermal behavior of TEWL above 40 °C whereas that below 40 °C may be closely related to the existence ratio of the Ort phase. However, several problems remain to be solved: (1) The behaviors of TEWL and XD in cooling are slightly different from those in heating, especially below 40 °C, (2) details of phase transition processes (from which phase to which phase) are still in debate, and (3) the TEWL measurement should be improved as discussed above. Here, we put forward a possible explanation of the thermal behavior of TEWL on the basis of the change in the intercellular lipid organization. However, many other explanations may be possible, considering that the skin SC is a multi-component complex system containing various materials other than lipid layers (e.g., keratin fibers). Thus, further study is needed to confirm the above speculations.
